# Contextual determinants of health behaviours in an aboriginal community in Canada: pilot project

**DOI:** 10.1186/1471-2458-12-952

**Published:** 2012-11-07

**Authors:** Pamela Joseph, A Darlene Davis, Ruby Miller, Karen Hill, Honey McCarthy, Ananya Banerjee, Clara Chow, Andrew Mente, Sonia S Anand

**Affiliations:** 1 Department of Medicine and Epidemiology, McMaster University, 1280 Main St, Hamilton, Ontario, L8S 4K1, Canada; 2 Six Nations Health Services, 1745 Chiefswood Road, Ohsweken, ON, N0A 1M0, Canada; 3 Population Health Research Institute, 237 Barton Street East, Hamilton, ON, L8L 2X2, Canada; 4 Chanchlani Research Centre, McMaster University, 1280 Main St. W., MDCL Rm. 3204, Hamilton, Ontario, L8S 4K1, Canada

**Keywords:** Obesity, Aboriginal health, Health behaviours, Environment design

## Abstract

**Background:**

Rapid change in food intake, physical activity, and tobacco use in recent decades have contributed to the soaring rates of obesity, type 2 diabetes and cardiovascular disease (CVD) in Aboriginal populations living in Canada. The nature and influence of contextual factors on Aboriginal health behaviours are not well characterized.

**Methods:**

To describe the contextual determinants of health behaviours associated with cardiovascular risk factors on the Six Nations reserve, including the built environment, access and affordability of healthy foods, and the use of tobacco.

In this cross-sectional study, 63 adults from the Six Nations Reserve completed the modified Neighbourhood Environment Walkability Scale (NEWS), questionnaire assessing food access and availability, tobacco pricing and availability, and the Environmental Profile of Community Health (EPOCH) tool.

**Results:**

The structured environment of Six Nations Reserve scored low for walkability, street connectivity, aesthetics, safety, and access to walking and cycling facilities. All participants purchased groceries off-reserve, although fresh fruits and vegetables were reported to be available and affordable both on and off-reserve. On average $151/week is spent on groceries per family. Ninety percent of individuals report tobacco use is a problem in the community. Tobacco is easily accessible for children and youth, and only three percent of community members would accept increased tobacco taxation as a strategy to reduce tobacco access.

**Conclusions:**

The built environment, access and affordability of healthy food and tobacco on the Six Nations Reserve are not perceived favourably. Modification of these contextual factors described here may reduce adverse health behaviours in the community.

## Background

Aboriginal people suffer a higher risk of developing obesity, type 2 diabetes and cardiovascular disease (CVD) compared to the general population [1-3]. A previous study we conducted on the Six Nations Reserve (SHARE-AP) demonstrated that community members have high rates of obesity, diabetes, hypertension, tobacco use, elevated cholesterol and CVD compared to European-origin Canadians [1]. In response to the high prevalence of obesity and related metabolic complications in adults, the SHARE-AP ACTION household-based intervention was implemented with the goal of changing health behaviours associated with weight gain. However after a six month intervention, only modest changes in dietary and physical activity practices were observed among intervention families [4]. Similarly, school and community-based interventions aimed at intensifying individual health behaviours among American Aboriginal communities have shown little impact on reducing body weight [5-8].

There is emerging evidence to support the influence of community level factors on health behaviors associated with CVD, such as diet, physical activity and tobacco use [9-12]. The built environment or “walkability” of a community is conceptualized as the extent to which environmental characteristics influence walking for leisure, exercise, recreation, travel and access to services [13]. In a systematic review of environmental determinants of health in adults, both availability of exercise equipment and connectivity of trails were associated with greater physical activity [14]. In Aboriginal communities, formative research has identified environmental factors, such as loose dogs, poor roads, safety and lack of facilities, as potential barriers to physical activity [4,5]. Similar environmental constraints may influence individual dietary behaviors. Aboriginal people living on remote reserves are exposed to high food prices and have limited access to healthy foods [15]. Aboriginal people also face increasing promotion and availability of energy-dense, nutrient poor products in comparison to fruits and vegetables [16]. While tobacco use has a traditional role in many Aboriginal cultures, expansion of tobacco production and easy access to cigarettes has resulted in a large proportion of adults using this highly addictive substance long-term [1]. It is well known that control of pricing and advertising is more effective for population control of tobacco use than are individualized smoking cessation programs [17]. However there is little regulation of tobacco access on most Aboriginal reserves in Canada.

The Six Nations Reserve in Brant County, Ontario spans approximately 18,000 hectares of land, and is located 25 km from the major city of Hamilton, between Brantford, Caledonia and Hagersville. The reserve is home to the largest population of First Nations in Canada, with over 12,000 Six Nations people [18,19]. Understanding the association of contextual factors and related CVD health behaviours in high-risk communities is important for the design of future interventions. Therefore the objective of this study was to characterize the contextual factors in the Six Nations community including the built environment, access and affordability of healthy food and tobacco. We hypothesize that contextual factors exist which constrain the ability of individuals to make and sustain health behaviour changes.

## Methods

Using a cross-sectional design, information was collected from individuals residing in the community to characterize community level factors of built environment, healthy food access and availability, and tobacco use among individuals from the Six Nations Reserve. The Research Ethics Board of McMaster University and Band Council of the Six Nations approved the study protocol and all study participants provided written informed consent. This study was funded by a grant from the Heart and Stroke Foundation of Ontario (Grant #PEA6547).

### Participants

Sixty-three individuals from the Six Nations Reserve were included in the present study. Study participants were previously enrolled in the SHARE-AP ACTION household-based randomized trial [4]. Eligibility and exclusion criteria are described elsewhere [4]. All 83 adult participants in SHARE-AP ACTION were mailed a letter describing the study, followed by telephone calls inviting them into the research unit to complete the assessment. The main reason cited for non-participation was being “too busy” at work or at home.

### Assessment and outcomes

Individuals completed questionnaires with a trained interviewer at the SHARE-AP research unit located on the reserve. Standardized tools were specifically tailored for the Aboriginal population living on the Six Nations Reserve to assess built environment, food availability, and tobacco availability, access and beliefs. A modified Neighbourhood Environment Walkability Scale (NEWS) was administered to assess built environment and individual perceptions of neighbourhood walkability. The scale is used to assess proximity and ease of access to community facilities, walking and cycling facilities, street connectivity, traffic and crime safety, aesthetics, and community satisfaction. Items are rated on a 4-point scale. NEWS has test-retest reliability, and has been validated against Geographic Information Systems, accelerometer and anthropometric measures in the United States [20-22]. This instrument has been used in rural communities in Taiwan and in an international prospective cohort study [23,24], and among an Indigenous population of New Zealand the Maori [25]. Food availability and access was assessed with a modified tool which was previously validated in a low socioeconomic sample in the United States [26]. Items include frequency and location of grocery shopping, food costs, availability and cost of fruits and vegetables on and off-reserve, and whether participants grow their own vegetables during the summer. An overall score was determined by an unweighted sum of five items. Perceptions of tobacco pricing and availability were measured using a Community Readiness Survey [27]. The perception score is scaled by the unweighted sum of the 8-items, which assess individual perceptions on tobacco use and accessibility among teenagers, problems with tobacco use among adults, willingness to increase tobacco tax, ban tobacco advertisements or enforce more laws on tobacco sales. The scale was found to have good construct validity, as scale scores were significantly associated with community readiness as evaluated by prevention planners [27]. The Environmental Profile of Community Health (EPOCH-2) tool measures community level determinants of cardiovascular risk factors and disease. This instrument has been tested in several communities in Canada, India, Columbia, China and Iran and was found to have good construct validity [28]. EPOCH-2 includes a survey of community awareness, attitudes and social norms given to all participants.

### Statistics

Statistical analysis was conducted using SPSS v11.0 software. All study variables were analyzed descriptively by percentage and means. Due to the limited statistical power we did not test the association between perceptions of the contextual environment and individual health behaviours.

## Results

Recruitment began in May 2008 and was completed in December 2008. Of the eighty-three participants approached from the previous SHARE-AP ACTION study, 75.9% (n=63) responded and were included in the study. Demographic characteristics of study participants are shown in Table 1. The mean age of participants was 42.3 (SD 10.7) years, with 41% of individuals making an annual income between $30,000 and $60,000. Thirty percent of study participants were current smokers.

**Table 1:** **Six nations participant demographics** (**N**=**63**)

**% Female**	**46(73**.**9%)**
**age mean**, **years(SD)**	**42**.**3(10**.**7)**
**Individual Income**, **N (%)**	
<$29,999	16 (27.6%)
$30,000 - $59,999	24 (41.4%)
>$60,000	18 (31.0%)
**Smoking Status**, **N (%)**	
Current	18 (29.5%)
Former/Never	43 (70.5%)

### Built environment (NEWS questionnaire)

Table 2 shows the mean and standard deviation of the walkability scores. Participants generally perceived the walkability of the Six Nations environment to be poor. For example, the score reported for walkability to community facilities, street connectivity, walking/cycling facilities, aesthetics, and pedestrian/traffic safety were all lower than the ‘midpoint’ score for each of the NEWS subscale components. Interestingly despite the unfavourable responses to the built environment, community satisfaction was reported to be high.

**Table 2:** **Mean** (**SD**) **subscale scores from the NEWS questionnaire**

**NEWS subscale**	**Six nations(N**=**63)**	**Possibility range**
Walkability to Community Facilities	8.9 (4.8)	7-42
Street Connectivity	6.7 (2.3)	4-16
Walking/Cycling Facilities	5.4 (2.7)	4-16
Aesthetics	7.5 (2.5)	5-25
Pedestrian/Traffic Safety	12.2 (2.3)	5-25
Crime Safety	16.4 (3.3)	5-25
Community Satisfaction	31.2 (6.8)	6-36

* Higher scores indicate a more favourable value of the Environmental characteristics.

### Food availability and affordability

Table 3 shows the participant responses for food availability and accessibility. The average individual reported spending $151 (SD $64.4) per week on groceries to feed their household. All individuals reported purchasing groceries off of the Six Nations reserve, which required an average of 5 (SD 2.56) trips per month. The majority of individuals reported that fruits and vegetables were affordable (89%) and available (98%) off of the reserve. Fewer people believed fruits and vegetables were affordable (76%) and available (67%) on the reserve. Twenty two percent grew their own fruits and vegetables in the summer.

**Table 3:** Fruits and vegetables accessibility and availability

**Fruits&vegetables accessibility and availability**	**N (%)or mean(SD)**
Average household money spent on Groceries per week	$150.66 (64.4)
Grocery Shopping done outside reserve	63 (100%)
Times per month to go Grocery Shopping	5 (2.56)
Grow own Vegetables & Fruits in Summer	14 (22.2%)
Fruits & Vegetables available inside the Reserve	42 (66.7%)
Fruits & Vegetables affordable inside the Reserve	48 (76.2%)
Fruits & Vegetables available outside the Reserve	62 (98.4%)
Fruits & Vegetables affordable outside the Reserve	56 (88.9%)

### Tobacco pricing and availability

Table 4 summarizes the responses for tobacco availability and accessibility. The majority of individuals on the Six Nations Reserve reported tobacco use to be a problem in their community (90.5%) and a problem among teenagers (90.5%). Most participants believed that there should be a ban on tobacco advertisement (74.6%) and greater law enforcement on tobacco sales (63.5%). However, only 3.2% of individuals were supportive of increased tobacco taxation. Three quarters of respondents indicated that teenagers can easily obtain tobacco from home; a majority believe that adults can easily buy tobacco for teens (87.3%), and teenagers can easily buy tobacco for themselves (66.7%).

**Table 4:** Tobacco pricing and availability factors

**Tobacco accessibility and availability variable**	**YES %**
Tobacco Use is a Problem in the Six Nations Community	90.5
There should be a ban on Tobacco Advertising	74.6
There should be more Law Enforcement on Tobacco sales	63.5
Tobacco Use Problem among Teenagers	90.5
Easy to Sneak Tobacco for Teenagers from Home	74.6
Easy for Adults to Buy Tobacco for Teenagers	87.3
Easy for Teenagers to Buy Tobacco	66.7
Willing to Pay More Tax on Tobacco	3.2

### Environmental profile of community health (EPOCH)

Participants were also asked whether they noticed tobacco and anti-tobacco advertisements in their community. (Table 5) Most reported seeing anti-smoking advertisements on television and radio (87.5%). 88.9% of individuals were aware of the laws requiring health warnings on cigarette packages. In terms of nutrition environmental factors, both junk food and healthy food advertisements were observed by study participants. Only 65% were aware of the official dietary guidelines, and 7.9% aware of the laws that lower fruit and vegetable tax (Table 6).

**Table 5:** Tobacco environmental factors

**Variable**	**YES %**
**Participant Observations**	
Tobacco Advertisements	
Posters	33.0
TV/Radio	12.0
Newspapers	34.9
Sports/Music	28.6
Anti Smoking Advertisements	
Posters	58.7
TV/Radio	87.5
Newspapers	63.5
**Participant Awareness**	
Support Programs to stop smoking	69.8
Bans of smoking in public	84.1
Bans on smoking advertising	84.1
Laws on health warnings	88.9

**Table 6:** **Nutrition environmental factors predicting daily fruit and vegetable consumption on six nations reserve from EPOCH**-**II** (**N**=**63**)

**Variable**	**YES %**
**Participant Observations**	
Junk Food Advertisements	
Posters	47.6
TV/Radio	69.8
Newspapers	63.5
Sports/Music	41.3
Healthy Food Advertisements	
Posters	49.2
TV/Radio	55.6
Newspapers	66.7
Sports/Music	33.3
**Participant Awareness**	
Official Dietary Guidelines	65.1
Laws that lower taxes on	7.9
fresh fruits/vegetables	

## Discussion

This study highlights several potential barriers toward adoption of healthy behaviours that exist in the Six Nations community. Specifically the built environment was viewed as being unfavourable to walking, access to healthy foods on the reserve was limited, and easy access to tobacco products was seen as problematic for the community.

While most respondents were satisfied living on the Reserve, participants identified the Reserve as being a difficult place in which to walk, having low street connectivity, poor aesthetics, and a higher potential for crime and traffic safety concerns. The ratings for neighbourhood walkability on the Six Nations reserve are very low in contrast to urban cities in Canada, where street connectivity, aesthetics, access to walking/cycling facilities and safety score above mid-point values on the NEWS questionnaire [29]. The NEWS questionnaire has been used to assess walkability in rural regions also, but not yet in Canada. It may be expected that walkability scores may be lower in rural compared to urban regions, although this is highly context dependent [23,24]. Enhancements to the walkability features of the Reserve may be an important target to increase physical activity among community members.

All participants purchased their groceries off of the Six Nations reserve, which is likely due to the greater availability and affordability of food. Six Nations respondents travel off of the Reserve more than 5 times per month in order to obtain a variety of food items, and spend approximately $151 per week on groceries alone, in contrast to the average total food expense of $140 per week in Ontario households [30]. In addition, Aboriginal households in Ontario are reported to have markedly lower household incomes ($46,865) in comparison to the Ontario median ($60,455), suggesting a greater burden of food costs is carried by Aboriginal families [31,32].

Overwhelmingly 90% of the community members reported tobacco use as being a problem in the Six Nations community especially among teenagers. Children and teenagers were perceived to have easy access to tobacco on the Reserve. Most participants are aware of smoking support programs, bans in public, advertisement laws, and health warnings on tobacco packaging. While most individuals indicated that bans on tobacco advertisements or increased law enforcement would be acceptable for reducing rates, only 3% were willing to tolerate increased taxation.

Prior studies of the association between contextual factors and health behaviours in non-Aboriginal communities such as physical activity, dietary intake, BMI, and tobacco use have produced mixed results. A recent systematic review of studies in which community-based interventions were initiated to improve community levels of physical activity reported inconsistent results, and emphasized the need for well-designed intervention studies [33]. Prior studies have reported that food availability and affordability [34] and presence of supermarkets [35] are associated with healthy food purchases, whereas difficulty accessing produce and high quality groceries promoted the consumption of fast food [36]. A recent systematic review of 28 studies, mostly from the United States showed that greater accessibility to supermarkets or less access to takeaway outlets was associated with a lower prevalence of overweight/obesity. However, no strong associations between the food environment and dietary intake were observed, with the exception of area-level deprivation where individuals who lived in low socioeconomic communities had a greater likelihood of having an obesogenic dietary intake [37]. Thus while community perceptions and contextual factors are associated with weight status, the association between the community environment and health behaviours such as physical activity, dietary intake, and BMI will require larger studies involving more communities [37]. For example, a fully powered study of the built environment on BMI would require inclusion of 12 Aboriginal communities, with at least 50 subjects per community to be able to detect a minimum 0.6 point difference in BMI between high versus low walkable communities (assuming an intraclass correlation of 0.09, and alpha of 0.05). Furthermore a recent review of intervention studies conducted among reserve dwelling Aboriginal people from the United States identified important components of successful community interventions designed to change health behaviours. These successful components include interventions targeted toward multiple levels including households, schools, community food suppliers as well as individuals, engaging and partnering with multiple community stakeholders and community members, and having an institutional base of support to ensure longevity of the health promotion program. They also highlight the importance of future research studies to study the effectiveness of community- based programs to change health behaviours and outcomes [38].

Current literature regarding effective tobacco-related community interventions in Aboriginal communities is sparse. Our observation that advertisements promoting smoking and easy access to smoking for adults and children on the Six Nations reserve, together with the previous observation we reported of the high prevalence of cigarette smoking, makes it highly likely that the two characteristics are linked. However data from individual studies vary. For example, a community and school-based intervention among Native American children from 27 elementary schools and 5 states showed no influence of a community-based intervention including classroom lessons, media, and information sessions on rates of cigarette use [39]. In a Cochrane review of community interventions targeting adult smoking by mass media, smoking policy, and smoke-free environments, had no effect on the prevalence of smoking [40]. The COMMIT randomized control trial of 11 communities in Canada and the United States did not change heavy smoker quit rates, but had a minimally significant influence on light-to-moderate smokers consuming less than 25 cigarettes per day [41].

There is strong evidence that increased taxation from a population perspective is associated with lower smoking rates, and it seems reasonable to assume that with increased taxation and decreased access to cigarettes, the rates of smoking on the Six Nations reserve would decline. The use of tobacco on the Six Nations reserve is complex. Registered Indians or bands on reserve are exempt from sales taxes for goods and services as outlined by section 87 of the Indian Act [42]. Although First Nations can implement taxes on tobacco sales under the First Nations Sales Tax (FNST), only nine communities have chosen to do so [43]. Many First Nations governments are dependent on federal fiscal transfers to account for more than 90 percent of their revenue. While local taxation of cigarettes could serve as a means for alternative and stable revenue of the elected Six Nations council, without a clear mechanism to enforce this policy, this strategy for tobacco control is not likely to be successful [44]. Increased price of tobacco is associated with lower tobacco use (price elasticity) in non-Aboriginal communities where smoking rates have decreased 2-4% for every 10% increase in price [45,46]. Opposition to tobacco taxation among First Nations is partly attributed to the strong historical, cultural, and economic ties to the tobacco industry. Successful tobacco control policies such as those advocated by the World Health Organization’s Framework Convention on Tobacco Control include common features such as banning all forms of promotion of tobacco products, price increases through taxation, strong prominent health warnings on packaging, banning sales to children, and enforcement of tobacco policy by law including implementation of quotas [47]. Presently on the Six Nations reserve these control policies do not exist – which does not bode well for the future health of this community given that the use of tobacco use among adults, pregnant mothers and children is substantially higher than in most other communities in Canada.

The World Health Organization and other health advocacy groups cite the aggressive global marketing of risky products and behaviors, particularly those targeting children and youth, as key factors in bringing tobacco, alcohol, and unhealthy processed foods into households worldwide [48]. In our survey, Six Nations community members recognized the excessive advertising of tobacco products and easy access for teenagers. However they did not embrace the idea that taxation may decrease use of tobacco. The community leadership in health must galvanize the community sentiment toward the adverse effects of tobacco on health, and persuade the local government to take a tough stand against the easy availability and access tobacco, especially among children and youth. The future health of Six Nations depends on the community’s involvement and ownership of new initiatives to target maladaptive health behaviors [48].

### Limitations

The small sample size of this study limited examination of direct associations between community level factors and individual behaviours. The evaluation tools we used may be too narrow to encompass all potential community level factors which impact on individuals health behaviours, as we relied on participants perception of their environment and not objective measures of the community. Other studies have shown that collection of both perception and objective measures of contextual factors enhances the ability to study influences of environment on health, and future studies should consider including both types of measures [49-51]. Furthermore our participants likely represented a more health conscious segment of the community (based on comparison of their higher household income and lower smoking rates to the general population) which may have influenced their responses to our questions. The effects of the off-reserve environment on Aboriginal health behaviours were not considered. In addition, the on-reserve environment is also extensive. The influence of contextual factors in schools and at the workplace would be relevant for analysis, as well as micro environmental components such as food quality, grocery store set-up, and restaurant menus [52]. Finally, the influence of multiple environments, including social, political and cultural factors, may overpower the independent influence of physical environment on behaviours. Inter-household sharing is a common practice in First Nations communities, suggesting the importance of studying the social environment on individual health behaviours [15].

This study highlights potential areas of interest for future study of contextual factors and community interventions. Our results suggest that interventions to improve reserve walkability, increase healthy food advertisements and nutrition education, and to reduce access and affordability of tobacco products may reduce the burden of chronic diseases faced by this community. Efforts should be made to find culturally appropriate community interventions that target health behaviours. Modifications to contextual factors have been attempted by other Aboriginal communities with successful intervention leading to increased access to fitness centers, use of walking trails, development of gardening programs, and improved food stores [53,54].

## Conclusion

The present study characterizes the contextual determinants of key health behaviours associated with cardiovascular risk on the Six Nations reserve. This study provides preliminary information to assist in the design, implementation and evaluation of contextual studies and interventions to determine if they are effective in improving health behaviours. It is likely that multiple prevention strategies including individualized programs as well as community interventions are needed to improve the future health of this community.

## Competing interests

Authors declare that there are no competing interests.

## Authors’ contribution

PJ, AB and AM assisted with the analysis, interpretation and drafting of the manuscript. HM, participated in data collection and provided comments of the manuscript drafts, CC assisted in the analysis of the EPOCH questionnaire data and provided comments on the manuscript drafts, RM helped to coordinate the study, and provided critical revisions on the manuscript drafts, SA conceived the study, and participated in analysis, interpretation and writing of the manuscript. All authors read and approved the final manuscript.

## Pre-publication history

The pre-publication history for this paper can be accessed here:

http://www.biomedcentral.com/1471-2458/12/952/prepub
